# Impact of Regulatory Approval Status on CADTH Reimbursement of Oncology Drugs and Role of Real-World Evidence on Conditional Approvals from 2019 to 2021

**DOI:** 10.3390/curroncol29110635

**Published:** 2022-10-26

**Authors:** Catherine Lau, George Dranitsaris

**Affiliations:** 1 158 Front Street East, Toronto, ON M5A 0K9, Canada; 2 Department of Public Health, Syracuse University, Syracuse, NY 13244, USA

**Keywords:** Health Canada, Canadian Agency for Drugs and Technology in Health (CADTH), real-world evidence (RWE), reimbursement, regulatory decision making

## Abstract

Real-world evidence (RWE) is health and outcomes data generated from a patient’s journey through the health care system or disease process (i.e., real-world data). RWE is now having an increasingly important role in regulatory/reimbursement decisions. This article examines reimbursement recommendations by the Canadian Agency for Drugs and Technology in Health (CADTH) on oncology drugs approved between 2019 and 2021. Oncology drugs with a Summary Basis of Decision (SBD) for original marketing approvals were used to generate a corresponding list of CADTH final clinical recommendations for review. Of the 45 oncology drugs approved by Health Canada, CADTH granted positive funding recommendations to all 11 drugs that had priority review approvals. Two of the 17 drugs with standard reviews did not file to CADTH and 3 received a negative recommendation. Of the 17 drugs with Notice of Compliance with Conditions (NOCc) status, three were not filed to CADTH and four were under active reviews. Of the ten completed NOCc reviews, all contained RWE from sponsors and six received a negative decision on their first review. No significant differences in review times were found between the three approval statuses. Regulatory approval status appeared to influence reimbursement outcomes in Canada and evaluation of 10 NOCc approvals provided little insight regarding robustness of RWE required for more favorable considerations.

## 1. Introduction

Real-world evidence (RWE) in medicine refers to evidence obtained from real-world data (RWD), which are health and outcome data generated from a patient’s journey outside the context of controlled clinical trials. RWE is generated by analyzing data obtained from patient registries, medical records, claims databases or in some cases hybrid trials, pragmatic trials, and late-phase trials [1]. The use of RWE in the post-authorization phase for safety signal detection and risk–benefit monitoring has been well recognized for decades [2]. It has been acknowledged that RWD generated in non-clinical trial settings can provide evidence that can either support, supplement or replace traditional clinical trial data for regulatory decision making [3]. With the advent of electronic medical databases and the development of sophisticated methodologies to analyze large datasets, discussions surrounding the use of real-world patient data in addition to clinical trials to support drug approval and reimbursement have gathered momentum. On 16 April 2019, Health Canada published the document “Optimizing the Use of Real World Evidence to Inform Regulatory Decision-Making”, acknowledging that the use of RWE for regulatory decisions is increasing globally in the assessment of drug safety, efficacy and effectiveness [4]. An accompanying document on the “Elements of Real World Data/Evidence Quality throughout the Prescription Drug Product Life Cycle” highlighted some of the standards determined by Health Canada to be important in supporting regulatory decision making. The document also identified that certain diseases/disorders (such as rare diseases) posed constraints on the conduct of randomized clinical trials (RCTs) and that studies based on RWE could be appropriate supporting evidence [5]. A strategy document published in March 2020 spelled out how Health Canada in collaboration with the Canadian Agency for Drugs and Technology in Health (CADTH) intended to operationalize the incorporation of RWD/E into decision making [6]. A more recent publication by CADTH outlined the formation of an RWE steering committee that will focus on orphan diseases as well as learning and collaboration with international groups [7].

Despite these promising public announcements, a recent publication suggested that Health Canada’s usage of RWE in recent regulatory decision making was low when compared with the usage by the U.S. Food and Drug Administration (FDA) and the European Medicines Agency (EMA) in oncology and orphan drugs. However, an increase in RWE use was observed in Notice of Compliance with Conditions (NOCc) reviews [8]. The study showed that the majority of conditional approvals were granted to oncology drugs due to the life-threatening and severe nature of these diseases. An in-depth review of Summary Basis of Decision (SBD) documents indicated that about 35% of oncology drug reviews mentioned the use of RWE to support efficacy. The publication indicated that Health Canada may have delegated the responsibility of reviewing RWE to CADTH so that RWE would have a more important role in reimbursement decisions [8]. The current review focuses on the use of RWE by CADTH, the federal agency responsible for making recommendations on drug and medical device reimbursement in Canada outside of the province of Quebec, which has its own reimbursement agency, the Institut national d’excellence en santé et en services sociaux (INESSS) [9].

CADTH published an environmental scan document in April 2018 that focused on the use of RWE in single-drug assessments [10]. This review article, which included the views of Health Canada and CADTH (based on surveys) and of other international agencies (based on available publications) concluded that, based on the evidence hierarchies utilized by regulatory agencies, the evidentiary value of RWE was rated to be lower than RCTs and could be used to supplement rather than to replace RCTs. The article also commented that in recent regulatory reviews, RWE was employed more frequently, especially for certain conditions such as drugs to treat cancer where testing for rare mutations has identified low-prevalence subgroups in which the conduct of large RCTs might be untenable.

Cancer is the leading cause of death in Canada [11,12]. The rapid development of targeted drugs as well as immuno- and cell therapies has dramatically improved the overall survival of cancer patients, delaying tumor progression and in some cases improving quality of life. To ensure these innovative therapies reach patients in the timeliest manner, regulatory agencies modified then-current regulations, with the FDA leading the process by creating new statuses such as the Breakthrough Therapy designation, Fast Track and more recently Real-Time Oncology Reviews [13]. Instead of creating new regulations, Health Canada has utilized existing regulations (both Priority Review and NOCc) to accomplish the same goal [14]. In 2021, over half of oncology drug approvals were granted NOCc status [8,15]. The approval time frame is shorter than for regular approvals and, more importantly, the data requirement is much lower with approvals being based on the promising nature of the medication for a life-threatening disease rather than on efficacy confirmed by appropriately sized randomized clinical trials.

Health Canada approvals allow new oncology drugs to be marketed in Canada, but reimbursement decisions by provincial and territorial publicly funded payers are made in light of reimbursement recommendations issued by the pan-Canadian Oncology Drug Review (pCODR), which operates within CADTH. Oncology drugs in Canada require a Health Technology Assessment (HTA) as part of this process [16,17]. The pCODR recommendations as to whether provincial and territorial public payers should reimburse these drugs are based on clinical benefit, patient values, cost effectiveness and feasibility of adoption within the Canadian health system. The accompanying cost-effectiveness model requires that the new medication be compared to the existing Canadian standard of care. For oncology drugs approved with NOCc status based on single-arm studies, finding a comparator is clearly a daunting task, especially for drugs treating new subsets of tumors identified by recently available molecular diagnostic tools. Real-world data collected from matched patients undergoing treatment with best or equivalent therapies were frequently used to generate RWE for cost-effectiveness models to support reimbursement [18].

Health Canada has elected to grant NOCc status to oncology drugs with single-arm studies with robust clinical outcomes and other promising characteristics. pCODR however requires RWE-facilitated comparator studies in order to build economic models for cost-effectiveness evaluation. Products approved with NOCc status were presumed to be at a disadvantage for reimbursement decisions, which led to companies delaying or avoiding reimbursement submissions to CADTH. A presentation by Lawrence Liberti [19], from the Centre for Innovation in Regulatory Science at a CADTH meeting held on 15 April 2019, compared regulatory approvals and reimbursement successes of conditional approvals between Health Canada, FDA, EMA and several European countries. Comparing regulatory approvals between Health Canada and FDA from 2015 to 2017, while 100% of FDA standard approvals were approved in Canada as standard Notices of Compliance with Conditions (NOCs), only 52% of FDA accelerated approvals were granted NOCc status by Health Canada. The percentage of CADTH negative recommendations for NOCc was 35.3%, much higher than the average of 5 countries in Europe, which was 16.3% [19]. This presentation unfortunately was not followed by a full-length publication, so the analysis would need to be confirmed through the peer review process. In addition, the presentation did not dissect out oncology products from other products. This article attempts to provide updated information on CADTH’s assessment of oncology products by evaluating how CADTH has used RWE in decision making and the impact of RWE on CADTH’s final recommendations on conditional approvals.

## 2. Methods

Review of databases:

*Health Canada Summary Basis of Decision (SBD) database:* Health Canada’s database containing summary reviews of all approvals from January 2019 to December 2021 was used as the anchor database [20] for all products studied in this review article. The review of the SBD database was performed as described previously [8]. Briefly, the database from 2019–2021 was downloaded into an Excel spreadsheet for abstraction. This previous publication covered SBDs published in the years 2020 and 2021 and the current review extends that period to cover 2019. The SBD products posted on the website between January 2019 and December 2021 were reviewed to search for anti-cancer drugs (which are termed “oncology products” in this review). Information recorded in SBD section (1) What was approved? Approval status, Standard reviews with Notice of Compliance (NOC), Notice of Compliance with conditions (NOCc) or Priority Review (PR) were noted and dates of submissions and approvals captured.

*CADTH Reimbursement Review Reports database:* The section of the CADTH website on reimbursement review reports [21] was downloaded into an Excel spreadsheet and drugs corresponding to Health Canada’s list of oncology drugs with SBDs published between January 2019 and December 2021 were abstracted. This review article focused on products with a CADTH assessment on their primary indications (first indication approved by Health Canada and first reviewed by CADTH). Final Clinical Guidance reports were reviewed for 8 NOCc products [22,23,24,25,26,27,28,29]. For two products that received their recommendations recently, selpercatinib [30] and tepotinib [31], the files on recommendations and draft recommendations were included in the review.

Review of CADTH Reports:

From the CADTH reimbursement review reports, the key milestone dates “Submission Date” and “Final Recommendation Issued” were used to calculate the time in review at CADTH and comparisons were made between oncology drugs approved by Health Canada with Standard, NOCc or PR status. The 5 attachment files within the CADTH review reports (1. Recommendations and Reasons; 2. Clinical and Pharmacoeconomic combined reports (separate Clinical and Economic guidance reports in earlier recommendations); 3. Patient Group Input Submission; 4. Clinician Input; and 5. Stakeholder Feedback on Draft Recommendations) were reviewed, and the determination was made that the assessment of fit-for-purpose RWE submitted by sponsors could be found in the Clinical and Pharmacoeconomic combined Reports or the Final Clinical Guidance reports. Section 1.2 (Key Results and Interpretations) and Section 7 (Supplemental Questions) were reviewed in detail to understand how CADTH critically appraised RWE information submitted by sponsors. CADTH’s comments on parameters critical for the validity of RWE (see Results) were extracted from CADTH’s clinical reviews on oncology products with NOCc approvals and comparisons were made between positive and negative reimbursement decisions.

Statistics:

All primary and secondary data collected were presented descriptively as means with appropriate measures of variance (95% CI). Comparisons of time in review between Health Canada and CADTH were undertaken using the unpaired *t*-test for unequal variances, using a one-sided test and a *p*-value of less than 0.025 for statistical significance. All of the statistical procedures were performed using Microsoft Excel 2010.

## 3. Results

**Figure 1:**
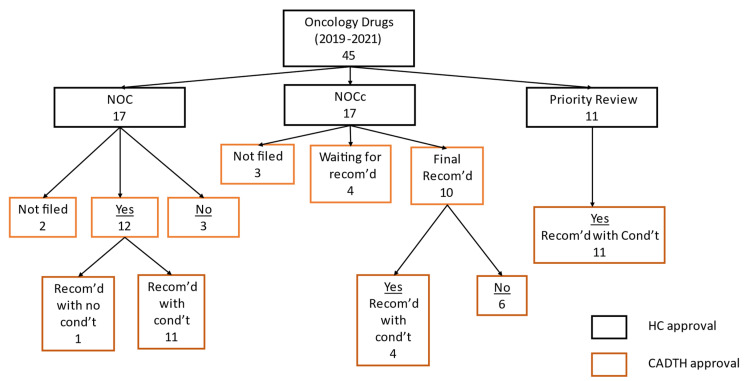
Canadian Agency for Drugs and Technology in Health (CADTH) approval disposition of submissions approved by Health Canada between 2019 and 2021.

*Health Canada oncology drug approval-status analysis based on SBD and submissions under review databases* [20]*:* From 2019 to 2021, the percentage of oncology drugs approved by Health Canada with Standard, NOCc and Priority approval status were 37.8%, 37.8%, and 24.4% respectively (Figure 1). Between 2019 and 2021, the percentage of oncology drugs receiving NOCc approvals was the same as for standard reviews and, when taken together with priority reviews, the percentage of oncology drugs with accelerated reviews was higher than standard reviews (Table 1 and Tables S1–S3). Mean days from submission to approval was shortest for Priority reviews (224, 95% CI (174, 274)), followed by Conditional reviews (285, 95% CI (206, 309)) and both were significantly shorter than Standard submission review time (368, 95% CI (346, 391)) (Table 1).

*CADTH oncology drug reimbursement decisions:* CADTH does not publish or commit to specific review timelines in their guidance document and, as can be seen in Table 2 and Tables S1–S3, the review time periods for oncology drugs with Standard, NOCc or Priority approvals from Health Canada were similar and not significantly different from each other (*p* > 0.1 for all comparisons).

Although CADTH issues three kinds of recommendations, “reimburse”, “reimburse with conditions” and “do not reimburse”, recently most positive reimbursement decisions were issued with conditions attached, which could be clinical or cost-related, so essentially two categories have dominated the reimbursement outcomes. When “reimburse” and “reimburse with conditions” were combined as positive decisions, CADTH’s positive reimbursement decisions for Health Canada Standard, NOCc and Priority reviews were 80%, 40% and 100% respectively (Table 2 and Tables S1–S3).

*Assessment of sponsor-submitted RWE by CADTH:* Submissions approved under Health Canada’s NOCc policy exhibited the lowest positive reimbursement decision level compared to Standard or Priority approvals. To investigate the impact of RWE on reimbursement decisions leading to Conditional approvals, CADTH’s critical appraisals of indirect evidence submitted by sponsors (Section 7 of the CADTH review report) on all NOCc products approved between 2019 and 2021 with completed CADTH reviews were descriptively quantified according to a variety of parameters (bias, heterogenicity, inability to adjust known or unknown confounders and other methodology flaws). These parameters were identified previously as having a considerable impact on the validity of the RWE evidence required to support efficacy or safety [32,33,34,35]. Table 3 and Table S4 list the parameters and provides examples from CADTH appraisals of NOCc products with positive (“reimburse with conditions”) or negative (“do not reimburse”) decisions. Examples of quotes abstracted directly from Section 7 of the final clinical reviews were similar between positive and negative reimbursement decisions (Table 3). According to CADTH, the RWE utilized in the comparator studies was less than optimal, with some of the most serious issues being bias (selection, information); heterogeneity related to trial populations, study design and cross trial comparisons; inability to adjust for confounding variables; prognostic variables and effect modifiers not being included; inappropriate methodology; and missing data requiring extensive imputations. All products regardless of the reimbursement decisions received similar levels of critique. Therefore, it was difficult to assess the evidence threshold required by CADTH for RWE utilization and its relationship to funding decisions.

## 4. Discussion

To our knowledge, this is the first article comparing how regulatory decisions influenced reimbursement decisions in Canada for recently approved oncology drugs and the role of RWE in the decision making process for NOCc approvals. As the guidance document was published by Health Canada in 2019 [5] and the collaboration with CADTH to operationalize the incorporation of RWE into decision making was announced in 2020 [6], the time period 2019–2021 was selected to optimally capture submissions that would have incorporated the elements of the guidance document. From 2019 to 2021, Health Canada reviewed 45 oncology drugs with 62.2% (Table 1) being accelerated approvals. The 37.7% NOCc approvals represented an almost a twofold increase compared to the 22% in a previous period (2010–2017) studied [36]. This dramatic increase in NOCc approvals for oncology drugs in recent years may have been influenced by regulatory actions adopted by both FDA and EMA to speed up approvals of promising anti-cancer treatments [37]. When the timelines between different approval statuses were examined, priority reviews had the shortest approval times followed by conditional approvals, with standard approvals taking significantly longer. Such time frames closely aligned with Health Canada’s published review policy [32]. These accelerated timelines of approvals benefited patients in Canada in accessing medications to treat life-threatening cancers. The COVID 19 pandemic did not appear to have impacted Health Canada oncology drug approval performance as the number of submissions/approvals from 2019–2021 was similar to 2017–2018 as assessed from reviewing Health Canada Drug Submission Performance Annual reports (direct request from publications-publications@hc-sc.gc.ca)

After regulatory approval is given by Health Canada, access to these therapies is largely determined by funding decisions made by the provinces and territories, which are heavily influenced by recommendations made by CADTH/pCODR, the federal agency in charge of reimbursement recommendations in Canada except Quebec. Previous publications [38,39] suggested that a negative CADTH recommendation generally precluded price negotiations and resulted in no public funding. The accelerated timelines achieved by Health Canada were not mimicked by CADTH as there were no significant differences in review times between oncology drugs with priority, conditional or standard reviews (Table 2). This is not surprising as a previous study that examined CADTH funding between 2011–2020 suggested that, although CADTH did have publicly stated target timelines to provide their recommendations, those targets were met only 50% of the time [33]. NOCc-approved products are considered to be “higher complexity” reviews by CADTH, so a shorter review time is unlikely [21]. In addition, rejection rates for reimbursement for NOCc approvals reached 60%, much higher than for standard (20%) and PR (0%) approvals.

Most of the NOCc approvals for oncology drugs were based on one or more single-arm clinical studies with small patient populations. For both NOC and NOCc drugs, CADTH submission guidelines required comparator studies (Canadian comparators preferred) as supplemental evidence and this information was generally utilized for the construction of an economic model for a cost-utility analysis [34]. For NOC products approved based on RCTs, network meta-analysis was recognized as an appropriate tool for indirect treatment comparisons. Population adjustment methods including Matching-Adjusted Indirect Comparison (MAIC) and Simulated Treatment Comparison (STC) were recommended for these cross-study indirect comparisons. MAIC applies propensity score reweighting and outcome regression to produce an indirect comparison in the aggregate data population. The novel aspect of MAIC and STC was to provide indirect comparisons when individual patient data (IPD) were only available for one arm of the comparison, which was often the case when companies would have IPD of their own clinical trials but not their competitors’ trials. “Anchored” indirect comparisons would be preferred over “unanchored” indirect comparisons, as evidence in the former is connected by a common comparator. A selected review of oncology drugs with NOC approvals showed CADTH critical appraisals of RWE differentiated positive from negative reimbursement decisions [40,41,42]. In the case of “unanchored” indirect comparisons used for NOCc, the evidence is disconnected due to the lack of a common comparator or single-arm studies [35,38,39,43]. Drugs approved based on RCT using a comparator or a placebo could potentially use the “anchored” MAIC methodology in a network meta-analysis (NMA) environment. Most of the oncology drugs approved under NOCc status based their pivotal clinical efficacy on single-arm trials and would inevitably be using “unanchored” MAIC, which required much stronger assumption based on population-adjusted indirect comparisons. For the comparison to be valid, absolute treatment effects were assumed to be constant at any given level of the effect modifiers and prognostic variables. In addition, all variables and effect modifiers were required to be known. These types of highly demanding assumptions were widely accepted as being difficult to meet. As reflected in CADTH critical appraisals, of the 10 NOCc oncology products reviewed between 2019 and 2021, none of the RWE submitted met the evidence threshold. When parameters critical to valid analyses of RWD (e.g., selection bias, heterogeneity in study populations, adjustment of confounding variables, missing data) were compared across the 10 products, no obvious differentiation could be identified (Table 3 and Table S4). Although most RWE submissions submitted by sponsors claimed that the indirect comparisons conducted yielded positive assessment outcomes over comparators, CADTH’s evaluation indicated that most of the studies were flawed and probably did not support the claims of the sponsors [22,23,24,25,26,27,28,29,30,31].

CADTH’s assessments were probably both candid and accurate, as instruments for indirect comparisons such as NMA, MAIC and STC did not apply well to single-arm studies. CADTH probably based its reimbursement decisions on clinical efficacy/safety, patient needs and physicians’ input. RWE was used as supportive evidence and probably had only minor impact on decision making. However, as more innovative treatments such as cell and gene therapies that can deliver transformative patient benefits become available, single-arm trials could become more of a norm for regulatory approvals [44]. CADTH and other HTA will need to explore existing [45] or emerging methodologies for evaluating the clinical and economic value of these agents. A recent discussion suggested that quantitative bias analysis (QBA) methods could be an option to handle missing data and residual confounding factors [46]. CADTH can explore collaborating with experts in academia, industry and regulatory agencies to assemble necessary expertise to ensure that valid RWE will be used for comparative effectiveness analysis and for the building of economic models. The recently assembled RWE Guidance Working Group by CADTH is definitely a step in the right direction [47].

Very few studies commented on use of RWE by CADTH in reimbursement decision making. A study by Anderson et al. [36] reviewing pCODR decisions on funding of oncology drugs between 2010 and 2017 indicated that conditional authorizations were associated with a recommendation against public funding by CADTH, which is consistent with our findings. Gotfrit et al. [33] while analyzing funding decisions by pCODR on different tumor types, reported that while positive funding decisions differed by tumor type, with the longest for colorectal cancer (*n* = 7) and shortest for neuroendocrine tumors (*n* = 3) (mean of 2541 days to 0 days), drug cost was not a significant factor for positive funding decisions. Consistent with our findings, the authors commented that the lack of transparent weighting schemes, objective thresholds for assessment and standards required for approval added a high degree of uncertainty to the decision making process. No comparisons were made between oncology drugs approved with standard or accelerated reviews such as PR or NOCc, or on the impact of RWE on reimbursement decisions. Another publication [18] reported on how the use of external comparators might affect health technology assessments based on single-arm trials. The report covered multiple HTAs including CADTH and suggested that submissions relying on single-arm trials had a high probability of receiving a positive CADTH recommendation if external comparators were used. The report did not publish product names or categories, so it unclear whether these comments applied to oncology drugs. Another review of pCODR Expert Review Committee (pERC) final recommendations made between January 2012 and May 2017 suggested that collection of RWE data can fill the gap of missing evidence. However, this review was presented as a vignette presentation [48] and no follow-up publication is available.

The strengths of this study are that it is the first comprehensive review on how regulatory decisions influenced reimbursement decisions in Canada for recently approved oncology drugs and the role of RWE in the decision making process for NOCc approvals. However, there are several limitations that need to be acknowledged. The key limitation is that the review was performed using publicly available information and sponsors’ submissions were not made public. Assessment of the appropriateness of using RWE for decision making by both Health Canada and CADTH was conducted based on the agencies’ reviews, which could be biased in their presentation of the evidence. The current paper however focused on the agencies’ opinion of the submissions and comparisons were made across products based consistently on the agencies’ published material, thus minimizing bias due to lack of sponsors’ information. In addition, not all NOCc approvals were submitted for CADTH review, and some of the approvals that were submitted may have been withdrawn, which would create a selection bias. As reported previously, the number of withdrawals was low [37] and one could speculate that submissions that were not filed or withdrawn were ones that sponsors considered to have a low probability of achieving a positive CADTH recommendation and would not have improved CADTH favorable rating for NOCc approvals.

Although the materials presented in the approval reports from both Health Canada and CADTH were reviewed, the focus was on those sections that included RWE. Since only these specific sections from the Health Canada and CADTH reports were selected and reviewed in detail, it is possible that some RWE information was missed in other sections of these reports. However, both Health Canada and CADTH reviews were template-driven and sections containing pivotal clinical information using RWE as supporting evidence were reviewed in detail. In light of this, the risk of having missed critical information relating to the use of RWE is believed to be small.

Information from CADTH critical appraisals was extracted for comparisons across oncology products that received NOCc approval. Criticisms by CADTH as assessed by the authors of this article did not differentiate between positive and negative funding decisions. However, these assessments could be biased, as the authors were not blinded to funding decisions before quantifying the appraisals. The authors however initiated the research with the preconceived hypothesis that differentiations would likely be found between positive and negative decisions, and were surprised with the actual findings. A lack of preconception would have minimized the bias but would not have been able to absolutely rule out the possibility of potential bias in the conclusion.

## 5. Conclusions

The findings of the current paper revealed that NOCc oncology drugs recently approved by Health Canada had the highest rate of negative reimbursement recommendations by pCODR. The results also suggested that RWE was used to a limited extent in supporting the data gaps. In cases where RWE was part of the submission, data quality issues did not ameliorate the uncertainty in the evidence package.

Therefore, the quality of RWE needs to be improved and one approach would be to create national standards for data quality. This may be achieved through the development of RWE consensus guidelines created by a key collaboration of all of the key stakeholders. Until such time, the quality of RWE generated to support NOCc drug submissions will remain suboptimal and the full potential of such a data source will never be realized. 

## Figures and Tables

**Table 1:** Health Canada review time for Standard, Priority and Conditional Reviews (see also Supplementary Tables S1–S3 for details).

Health Canada Review of Drugs with SBD Published between 2019 and 2021	Number of Submissions *n* = 45 % (*n*)	Days in Review: Mean (95% CI)	Conditional and Priority Review Times as Compared to Standard Review Times	Comparing Priority Review Time as to Conditional Review Time
**Standard review (NOC)** **% (*n*)**	37.78 (17)	368 (346, 391)		
**Conditional Review (NOCc)** **% (*n*)**	37.78 (17)	285 (206, 309)	*p* < 0.001	
**Priority Review (PR)** **% (*n*)**	24.44 (11)	224 (174, 274)	*p* < 0.001	*p* = 0.025

**Table 2:** CADTH review time and Reimbursement Decisions for Standard, Conditional and Priority reviews (see also Supplementary Tables S1–S3 for details).

CADTH Review of Oncology Drugs (with Final Recommendations Only)	Standard Approvals (*n* = 15) ^a^	Conditional Approvals NOCc (*n* = 10) ^b^	Priority Reviews PR (*n* = 11)	Conditional Review Times and Priority Review Times Compared to Standard Review Times
**Review time (days)** **Mean (95% CI)**	238 (216, 260)	240 (258, 188)	252 (223, 308)	Conditional to Standard: *p* = 0.98 Priority to standard: *p* = 0.57
**Reimburse without conditions** **% (n)**	6.7 (1)	0	0	NA
**Reimburse with conditions** **% (n)**	73.3 (11)	40 (4)	100 (11)	NA
**Do not reimburse** **% (n)**	20 (3)	60 (6)	0	NA

**^a^** 2 products did not file a submission to CADTH; **^b^** 3 products did not file and 4 products are under active review.

**Table 3:** Examples of criticisms from CADTH appraisals of Health Canada Conditional approvals (NOCc) approvals (see also Supplementary Table S4).

Parameters with Impact on RWE Validity	Examples Extracted from CADTH Review Reports	Decisions on Reimbursement [Ref]
*Bias*		
**Selection Bias**	High risk of selection bias owing to the retrospective nature of the historical comparator.	+ve [24]
**Information Bias**	The bias resulting from missing covariates is very difficult to quantify, and as a result, it is unclear what impact the missing covariates have on the results of the MAICs.	−ve [23]
**Endpoint Assessment Bias**	There is also a potential measurement bias due to differences in the frequency and conduct of disease assessments in clinical practice versus the trial setting.	+ve [30]
*Heterogenicity*		
**Study populations/trials/across-study comparisons**	These ITCs have a number of limitations that impact their internal and external validity, such as not being able to comprehensively assess the clinical heterogeneities across the included individual studies and their influence on the study results due to the lack of certain patient characteristics, uncertainty still exists on the treatment effect of selpercatinib despite of various adjustments, and generalizability of the study findings to patients with RET fusion-positive could be limited.	+ve [30]
	There was limited assessment and reporting of clinically important heterogeneity, and the statistical analyses completed are unlikely to have accounted for all major differences.	−ve [31]
*Unresolved Confounders*		
**Inability to adjust confounders, measured or unmeasured, prognostic variables and effect modifiers not included**	The major concerns with the submitted report are related to the quality of the analysis, limited control of prognostic factors and effect modifiers, and the heterogeneity of the evidence used.	+ve [29]
	However, it is not clear if the underlying assumption of the unanchored MAIC that all effect modifiers and prognostic factors have been accounted for was accomplished.	−ve [23]
	None of the articles retrieved through the literature search spoke directly about the prognostic relevance of the specific gene fusion. All primary studies retrieved through the literature search were retrospective in design. In cases where presence of the specific gene fusion was verified, sample sizes were small, making the generalizability of findings difficult to determine.	−ve [30]
*Methodology issues*		
**Use of inappropriate methods**	No statistical analyses were performed (e.g., multivariate regression model analyses) to identify a subset of variables most predictive of outcome to include for matching. Further, it is unknown how missing data on variables used for matching were handled in the analysis.	+ve [25]
	These limitations, combined with the flaw in the presentation of the methodological quality of the included studies, limits the overall confidence in the results of the methodological quality of the included studies, limits the overall confidence in the results of this review.	−ve [28]
*Data issues*		
**Missing data and extensive imputation**	Fewer than 25% of the remaining adherent patients continued the assessment after week 29. When the data was presented in linear plots, there appears to be higher scores in the [treated] groups and scores remained flat in the BR group, however the significant amount of missing data limits confidence in this analysis.	+ve [29]
	which implies potential bias due to the need to rely on multiple imputation methods, increasing the uncertainty in effect estimates.	−ve [23]

## Data Availability

Not applicable.

## Supplementary Materials

The following supporting information can be downloaded at: https://www.mdpi.com/article/10.3390/curroncol29110635/s1, Table S1. Health Canada and CADTH review times and reimbursement decisions for oncology products with standard approvals (NOC) between 2019 and 2021; Table S2. Health Canada and CADTH review times and reimbursement decisions for oncology products with conditional approvals (NOCc) between 2019 and 2021; Table S3. Health Canada and CADTH review times and reimbursement decisions for oncology products with Priority approvals (PR) between 2019 and 2021; Table S4. Examples of critical appraisals of Health Canada Conditional approvals (NOCc) by CADTH reviewers.
