# Supplementary material for: Impact of Regulatory Approval Status on CADTH Reimbursement of Oncology Drugs and Role of Real-World Evidence on Conditional Approvals from 2019 to 2021
Source: Curr Oncol. 2022 Oct 26;29(11):8031–42. doi: 10.3390/curroncol29110635 (PMC9689244; doi:10.3390/curroncol29110635)
Supplement: Supplementary file 1 [file curroncol-29-00635-s001.zip › curroncol-1932366-supplementary.pdf]

Supplementary Materials for "Impact of Regulatory Approval Status on CADTH Reimbursement of Oncology Drugs and Role of Real-World Evidence on Conditional Approvals from 2019 to 2021" by C. Lau and G. Dranitsaris

Table S1. Health Canada and CADTH review times and reimbursement decisions for oncology products with standard approvals (NOC) between 2019 and 2021

Drugs (generic names) Indicated for Tumor Type	Summary Basis of Decision (SBD) publication date	Health Canada submission date	Health Canada approval date	Time in review by Health Canada	CADTH submission date	CADTH recommendation date	Time in review by CADTH	CADTH recommendations
Binimetinib Melanoma	6/8/2021	03/20/2020	3/02/2021	347	12/16/2020	7/8/2021	204	Reimburse with cond't
Encorafenib CRC	6/8/2021	03/20/2020	3/02/2021	347	12/16/2020	7/8/2021	204	Reimburse with cond't
Isatuximab MM	12/8/2020	06/28/2019	4/29/2020	306	8/17/2020	4/1/2021	227	Reimburse with cond't
Fedratinib MF	10/22/2020	07/19/2019	7/27/2020	374	11/5/2020	6/21/2021	228	Reimburse with cond't
Sonidegib BCC	8/11/2020	07/04/2019	6/12/2020	344	6/19/2020	04/29/2021	314	Do not Reimburse
Glasdegib AML	7/10/2020	03/15/2019	4/2/2020	384	5/6/2020	1/8/2021	247	Do not Reimburse
Gemtuzumab ozogamicin AML	7/10/2020	12/19/2018	11/28/2019	344	8/9/2019	2/4/2020	179	Reimburse
Darolutamide Prostate Cancer	6/29/2020	03/27/2019	2/20/2020	330	8/27/2019	4/22/2020	239	Reimburse with cond't
Neratinib Breast Cancer	1/29/2020	06/21/2018	7/16/2019	390	4/18/2019	12/5/2019	231	Do not reimburse
Talazoparib Breast Cancer	1/21/2020	9/28/2018	9/06/2019	343	Not filed			
Acalabrutinib MCL	1/13/2020	03/15/2018	8/23/2019	526	4/7/2020	11/17/2020	222	Reimburse with cond't
Dinutuximab NET	5/6/2019	12/14/2017	11/28/2018	349	11/23/2020	7/23/2021	242	Reimburse with cond't

Decitabine<br>MDS	6/25/2019	12/21/2017	1/21/2019	396	10/9/2020	9/22/2021	348	Reimburse with cond't
Abemacicib<br>Breast Cancer	9/26/2019	04/06/2018	4/5/2019	364	12/3/2018	7/5/2019	214	Reimburse with cond't
Dacomitinib<br>NSCLC	10/22/2019	03/16/2018	2/26/2019	347	9/18/2018	5/31/2019	255	Reimburse with cond't
Niraparib<br>Ovarian Cancer	11/19/2019	05/31/2018	6/27/2019	392	9/21/2020	4/21/2021	212	Reimburse with cond't
Decitabine<br>MDS	12/11/2019	06/28/2018	7/11/2019	378	Not filed			

Abbreviations:

CRC: Colorectal Cancer

MM: Multiple Myeloma

MF: Myelofibrosis

BCC: Basal Cell Carcinoma

AML: Acute Myeloid Lymphoma

MCL: Mantle Cell Lymphoma

MDS: Myeloid Dysplastic Syndrome

Table S2. Health Canada and CADTH review times and reimbursement decisions for oncology products with conditional approvals (NOCc) between 2019 and 2021

Drugs (generic names) Indicated for Tumor Type	Summary Basis of Decision (SBD) publication date	Health Canada submission date	Health Canada approval date	Time in review by Health Canada	CADTH submission date	CADTH recommendation date	Time in review by CADTH	CADTH recommendations
Sotorasib NSCLC	12/7/2021	1/14/2021	10/22/2021	239	Not filed			
Infigratinib CC	11/30/2021	11/30/2020	9/27/2021	301	Not filed			
Pralsetinib NSCLC	11/26/2021	9/8/2020	6/30/2021	295	3/9/2022	active		
Tafasitamab DLBCL	11/25/2021	12/4/2020	8/19/2021	258	11/19/2021	active		
Idecabtagene vicleucel MM	11/22/2021	9/17/2020	5/26/2021	251	12/16/2020	11/12/2021	331	Do not reimburse
Selpercatinib NSCLC and Thyroid Cancer	11/9/2021	9/10/2020	6/15/2021	278	10/29/2021	5/5/2022	188	Reimburse with cond't
Tepotinib NSCLC	10/21/2021	7/31/2020	5/27/2021	300	8/30/2021	8/24/2022	360	Do not reimburse
Trastuzumab deruxtecan Breast Cancer	7/9/2021	7/24/2020	4/15/2021	265	3/23/2022	active		
Polatuzumab vedotin DLBCL	10/8/2020	10/04/2019	7/9/2020	279	9/29/2020	4/21/2021	204	Reimburse with cond't
Entrectinib ECT	7/16/2020	5/7/2019	2/10/2020	279	1/25/2022	active		
Brigatinib NSCLC	1/28/2019	10/17/2017	7/26/2018	282	12/05/2018	8/01/2019	235	Do not reimburse
Pralatrexate NSCLC	4/29/2019	7/18/2017	10/26/2018	465	6/1/2018	4/4/2019	307	Reimburse with cond't

Lorlatinib NSCLC	7/18/2019	4/26/2018	2/22/2019	302	6/11/2019	1/30/2020	233	Do not reimburse
Enasidenib AML	8/19/2019	6/15/2018	2/6/2019	236	4/19/2019	10/31/2019	195	Do not reimburse
Cemiplimab-rwlc CSCC	8/27/2019	07/27/2018	4/10/2019	257	7/9/2019	1/22/2020	197	Reimburse with cond't
Larotrecinib NTRK mutated tumor	12/18/2019	9/18/2018	7/10/2019	295	11/16/2020	9/13/2021	301	Do not reimburse
Erdafitinib Bladder Cancer	1/28/2020	2/8/2019	10/25/2019	259	Not filed			

Abbreviations:

NSCLC: Non-Small Cell Lung Cancer

CC: Cholangiocarcinoma

MM: Multiple Myeloma

DLBCL: Diffused Large B Cell Lymphoma

ECT: Extra Cranial solid Tumor

AML: Acute Myeloid Leukemia

CSCC: Cutaneous Squamous Cell Carcinoma

NTRK: Neurotrophic Receptor Tyrosine Kinase

Table S3. Health Canada and CADTH review times and reimbursement decisions for oncology products with Priority approvals (PR) between 2019 and 2021

**Drugs (generic names) Indicated for Tumor Type**	**Summary Basis of Decision (SBD) publication date**	**Health Canada submission date**	**Health Canada approval date**	**Time in Review by Health Canada**	**CADTH submission date**	**CADTH recommendation date**	**Time in review by CADTH**	**CADTH recommendations**
Brexucabtagene autoleucel MCL	12/6/2021	11/13/2020	6/8/2021	207	12/18/2020	8/6/2021	231	Reimburse with cond’t
sacituzumab govitecanhziy Breast Cancer	12/23/2021	01/25/2021	9/24/2021	242	6/30/2021	1/22/2022	206	Reimburse with cond’t
Tucatinib Breast Cancer	10/1/2020	01/20/2020	6/3/2020	137	3/26/2021	11/1/2021	220	Reimburse with cond’t
Ipretinib GST	9/22/2020	12/23/2019	6/19/2020	179	10/15/2021	4/14/2022	181	Reimburse with cond’t
Decitabine and cedazuridine MDS	9/21/2020	12/31/2019	7/7/2020	189	10/9/2020	9/22/2021	348	Reimburse with cond’t
Axicabtagene ciloleucel DLBCL	8/15/2019	07/19/2018	2/13/2019	209	10/25/2018	8/15/2019	294	Reimburse with cond’t
Zanubrutinib WM	7/15/2021	08/13/2020	3/01/2021	200	5/21/2021	12/21/2021	214	Reimburse with cond’t
Lutetium Lu177 dotatate NET	5/29/2019	06/18/2018	1/9/2019	205	7/30/2018	8/1/2019	367	Reimburse with cond’t
Giltertinib AML	3/23/2019	5/15/2019	12/23/2019	222	10/19/2019	5/20/2020	214	Reimburse with cond’t
Cabozantinib NSCLC	2/12/2019	06/05/2017	9/14/2018	466	9/17/2018	2/20/2019	156	Reimburse with cond’t
Tisagenlecleucel RCC	2/8/2019	02/04/2018	9/5/2018	208	2/9/2018	1/15/2019	340	Reimburse with cond’t

Abbreviations:

MCL: Mantle Cell Lymphoma

GST: Gastrointestinal Stroma Tumor

MDS: Myeloid Dysplastic Syndrome

DLBCL: Diffused Large B Cell Lymphoma

WM: Waldenstrom Macroglobulinema

NET: Neuro Endocrine Tumor

AML: Acute Myeloid Leukemia

NSCLC: Non-Small Cell Lung Cancer

RCC: Renal Cell Carcinoma

Table S4. Examples of critical appraisals of Health Canada Conditional approvals (NOCc) by CADTH reviewers

**Parameters important for the evaluation of RWE to be incorporated into decision-making**	**Examples from the CADTH "Clinical Review" for "Reimburse with Conditions" for NOCc approvals (N=4)**	**Examples from the CADTH "Clinical Review" for "Do Not Reimburse" for NOCc approvals (N=6)**
Bias:<br>- Selection bias/information bias/endpoint assessment bias	- the most significant of these included the high risk of selection bias owing to the retrospective nature of the historical comparator data<br>- Thus, the prognostic factors missing from the models may have had an influence on the outcomes of interest and the reported estimates therefore may be biased.<br>- These differences are largely unaccounted for in the methodology and may produces systematic differences between populations and introduce bias in the analyses<br>- There is also a potential measurement bias owing to differences in the frequency and conduct of disease assessments in clinical practice versus the trial setting	- The results may be biased due to unmeasured baseline characteristics and failure to conduct a quality assessment of the included studies<br>- Bias due to imbalance in unmeasured confounders is a potential limitation to these results<br>- The bias resulting from missing covariates is very difficult to quantify, and as a result, it is unclear what impact the missing covariates have on the results of the MAICs.
Heterogenicity<br>- Study population/trials/across studies	- The studies differed from each other in notable ways. Most notable to the heterogeneity of the studies was the designs, inclusion/exclusion criteria, and the median follow-ups.<br>- These ITCs have a number of limitations that impact their internal and external validity, such as not being able to comprehensively assess the clinical heterogeneities across the included individual studies and their influence on the study results due to the lack of reporting certain patient characteristics, uncertainty still exists on the treatment effect	- Differences that arose during study design and when combining patients across databases (i.e., selecting patients from different databases) may result in the introduction of clinical heterogeneity that cannot be accounted for in propensity score modelling.<br>- there was limited assessment and reporting of clinically important heterogeneity, and the statistical analyses completed are unlikely to have accounted for all major differences.

Inability to adjust of cofounders, measured or unmeasured, prognostic variables and effect modifiers not included	- due to the lack of reporting certain patient characteristics, uncertainty still exists on the treatment effect of X despite of various adjustments, and generalizability of the study findings to patients<br>- A number of limitations that impact their internal and external validity, such as not being able to comprehensively assess the clinical heterogeneities across the included individual studies and their influence on the study results due to the lack of reporting certain patient characteristics, uncertainty still exists on the treatment effect<br>- the submitted reports are related to the quality of the analysis, limited control of prognostic factors and effect modifiers, and the heterogeneity of the evidence used<br>- A MAIC adjusts for trial differences in known prognostic factors or treatment effect modifiers; however, it does not account for unknown cross-trial differences that may be present. Consequently, treatment effect estimates obtained by the MAIC are still susceptible to bias resulting from unknown confounding<br>- While a targeted literature search was performed for prognostic factors, no effect modifiers were considered in the analysis. In the Core model, the sponsor only included prognostic factors that were reported as statistically significant in at least one of the studies identified in the Systematic Literature Research (SLR). Prognostic factors found to be non-statistically significant were included in the extended model. In order to obtain an unbiased estimate of differences in the treatment effects, all prognostic factors and effect modifiers for a given outcome must be adjusted for in the model.	- however, it is not clear if the underlying assumption of the unanchored MAIC that all effect modifiers and prognostic factors have been accounted for was accomplished.<br>- None of the articles retrieved through the literature search spoke directly about the prognostic relevance of the specific gene fusion. All primary studies retrieved through the literature search were retrospective in design. In cases where presence of the gene fusion were verified, sample sizes were small, making the generalizability of findings difficult to determine<br>- the lack of adjustment for all important potential confounders in the analyzes. In view of the substantial uncertainty in the ITC results, pERC could not draw any conclusions pertaining to the efficacy<br>- inability to adjust for all potential confounders and prognostic variables and use of inappropriate analysis methods for MAIC (e.g., not providing residual bias estimates for MAICs).<br>- Imbalance remained after matching for the cytogenetic risk profile. Patients in the France chart review study appear to have a better cytogenetic risk profile than the trial population, after matching. The results of the analysis can be misleading, given that the ATU represents the effect of the drug

Methodology<br>Use of inappropriate methods	- No statistical analyses were performed (e.g. multivariate regression model analyses) to identify a subset of variables most predictive of outcome to include for matching. Further, it is unknown how missing data on variables used for matching were handled in the analysis<br>- due to the limitations identified, the findings from the MAIC were inconclusive because the assumptions used for the unanchored analyses are impossible to meet and present an unknown amount of bias in the unanchored estimate	- These limitations, combined with the flaw in the presentation of the methodological quality of the included studies, limits the overall confidence in the results of the methodological quality of the included studies, limits the overall confidence in the results of this review<br>- Any potential risks of bias of the included data sources (i.e., methodological limitations) were not assessed and not reported.<br>- The NMA is inherently flawed given the use of unanchored MAIC data to create "virtual studies" to represent head-to-head trials within the networks
Data missing with extensive imputation	- Fewer than 25% of the remaining adherent patients continued the assessment after week 29. When the data was presented in linear plots, there appears to be higher scores in the treated groups and scores remained flat in the BR group, however the significant amount of missing data limits confidence in this analysis.	- In addition, the indirect comparisons may have been biased by the differential distribution of invalid or missing data between the V clinical trial and retrospective datasets<br>- which implies potential bias due to the need to rely on multiple imputation methods, increasing the uncertainty in effect estimates.
